# Clinical characteristics and outcomes of immune checkpoint inhibitor-induced pancreatic injury

**DOI:** 10.1186/s40425-019-0502-7

**Published:** 2019-02-06

**Authors:** Hamzah Abu-Sbeih, Tenglong Tang, Yang Lu, Selvi Thirumurthi, Mehmet Altan, Amir A. Jazaeri, Ramona Dadu, Emmanuel Coronel, Yinghong Wang

**Affiliations:** 1 Departments of Gastroenterology, Hepatology & Nutrition, The University of Texas MD Anderson Cancer Center, Houston, TX USA; 2 Department of Minimally Invasive Surgery, The Second Xiangya Hospital of Central South University, Changsha, Hunan People’s Republic of China; 3 Departments of Nuclear Medicine, The University of Texas MD Anderson Cancer Center, Houston, TX USA; 4 Departments of Thoracic/Head and Neck Medical Oncology, The University of Texas MD Anderson Cancer Center, Houston, TX USA; 5 Departments of Gynecologic Oncology and Reproductive Medicine, The University of Texas MD Anderson Cancer Center, Houston, TX USA; 6 Departments of Endocrine Neoplasia and Hormonal Disorders, The University of Texas MD Anderson Cancer Center, Houston, TX USA

**Keywords:** Immunotherapy, Immune checkpoint inhibitor, Cytotoxic T-cell lymphocyte-4 (CTLA-4), Programmed death-1 and ligand-1 (PD-1/L1), Pancreatitis, Lipase elevation, Pancreatic injury, Pancreatic adverse events

## Abstract

**Background:**

Immune checkpoint inhibitor (ICI)-induced pancreatic injury (ICIPI) is not well documented in the literature. We aimed to describe the clinical characteristics and outcomes of patients who developed ICIPI.

**Methods:**

We reviewed the medical records of consecutive patients who had a confirmed diagnosis of ICIPI (Common Terminology Criteria for Adverse Events grade ≥ 3 lipase elevation with or without clinical symptoms) from April 2011 through April 2018.

**Results:**

Among the 2,279 patients received ICI and had lipase values checked thereafter, 82 (4%) developed ICIPI. Overall, 65% of patients received inhibitors of programmed death protein-1 or its ligand. Compared with asymptomatic presentation, patients who had clinical symptoms of pancreatitis (*n* = 32) had higher levels of lipase (*P* = 0.032), more frequent imaging evidence of pancreatitis (*P* = 0.055), and more frequent hospitalization (*P* < 0.001) and received intravenous fluids (*P* < 0.001) and steroids more frequently (*P* = 0.008). Twelve patients (15%) developed long-term adverse outcomes of ICIPI; three had chronic pancreatitis, four had recurrence of ICIPI, and six had subsequent diabetes. Among 35 patients who resumed ICI therapy, four (11%) had recurrence of lipase elevation. Logistic regression revealed that smoking and hyperlipidemia were associated with increased risk for long-term adverse outcomes of ICIPI, and intravenous fluids were associated with reduced risk. Patients who resumed ICI therapy survived longer than patients who discontinued ICI therapy permanently, statistically not significant (*P* = 0.0559). Patients who developed long-term adverse outcomes of ICIPI survived significantly longer than those who did not (*P* = 0.0295). The highest proportion of patients (6/21, 29%) developed long-term adverse outcomes of ICIPI was among those without typical symptoms of pancreatitis, continued ICI therapy after ICIPI, and did not receive intravenous fluids.

**Conclusion:**

ICIPI can present as typical acute pancreatitis, with risk of the development of a pseudocyst, diabetes, and chronic pancreatitis. ICI resumption after ICIPI may lead to recurrence of lipase elevation without increased risk of long-term adverse outcomes, and can increase survival duration. Intravenous fluids may prevent long-term adverse outcomes, but steroids do not appear to affect outcomes of ICIPI. Asymptomatic ICIPI presentation may lead to undertreatment of ICIPI owing to underestimation of its degree, and therefore, intravenous fluid administration could potentially could potentially be benificial to prevent long-term adverse outcomes even in asymptomatic patients.

**Electronic supplementary material:**

The online version of this article (10.1186/s40425-019-0502-7) contains supplementary material, which is available to authorized users.

## Introduction

Immune checkpoint inhibitors (ICIs), including cytotoxic T-cell lymphocyte-4 (CTLA-4) and programmed death-1 (PD-1)/ligand-1 (PD-L1) inhibitors, are effective in the treatment of a rapidly increasing number of advanced cancer types, including malignant melanoma, non-small cell lung cancer, head and neck cancers, and genitourinary and hematologic malignancies [1–9]. Currently, in the United States, 600,000 patients could benefit from ICI therapy and the number is expected to increase [10]. The overall survival rate of patients with advanced cancers has improved significantly during the past decade as a consequence of these ICI agents [11–13].

Nonetheless, ICI therapy has some drawbacks, in particular, immune-related adverse events (irAEs). IrAEs can affect almost any organ system and range from mild self-limiting symptoms to severe life-threatening events. The most commonly affected organ systems are dermatologic, gastrointestinal, hepatic, pulmonary, and endocrine systems. [14] The evaluation and management of these common irAEs have been studied extensively, and favorable cancer treatment outcomes have been reported in some cases [15–19]. Lipase elevation is an uncommon irAE, with limited information available in published studies [20–24]. One of the obstacles confronting the investigation of ICI-induced pancreatic injury (ICIPI), in addition to the very small number of patients owing to rare occurrence, is the involvement of various factors as potential causes for the elevated serum lipase. Therefore, published evidence regarding the incidence and clinical characteristics of irAEs affecting the pancreas (i.e., ICIPI) is sparse.

The resemblance between ICIPI and traditional acute pancreatitis that presents with clinical symptoms, in addition to elevated serum lipase and imaging abnormalities, has not yet been studied. ICIPI is usually asymptomatic with normal imaging findings of the pancreas, and the elevated serum lipase is detected incidentally [25]. Moreover, the clinical and imaging presentation of ICIPI is nonspecific and can be attributed to several causes. Thus, current guidelines for the management of ICIPI are based on scant evidence [26–28]. Furthermore, there is no clear evidence on the impact of ICI therapy discontinuation or continuation on the pancreas after the occurrence of ICIPI.

Hence, in this retrospective case series, we aimed to describe the clinical characteristics, treatment, and outcomes of patients who developed ICIPI at a tertiary cancer center.

## Materials and methods

### Study design and population

We conducted a retrospective, descriptive study of adult patients who received ICI therapy at The University of Texas MD Anderson Cancer Center and developed grade ≥ 3 elevation of the serum lipase according to Common Terminology Criteria for Adverse Events (CTCAE) v4.03 from April 2011 through April 2018. Grade 3 is defined as lipase elevation of > 2 times the upper limit of normal (60 U/L in our laboratory). The study was approved by the Institutional Review Board at MD Anderson. Included patients met all of the following criteria: (1) aged 18 years or older, (2) received ICI therapy, and (3) developed grade ≥ 3 elevation of serum lipase during or after ICI therapy within 6 months. After a comprehensive medical chart review, we excluded patients who developed acute pancreatitis due to other reasons, such as endoscopic retrograde cholangiopancreatography, gallstone or biliary obstruction, alcohol, and other non-ICI drugs that have known risk of pancreatic toxicity.

### Clinical data

Collected data consisted of patient demographics, comorbidities, oncology history, ICI regimen, and non-pancreatic irAEs. Patient demographics consisted of age, sex, and race/ethnicity. Smoking history, alcohol consumption, type II diabetes mellitus, drug allergy, prior history of pancreatitis, and pancreatic metastasis were considered and recorded as baseline risk factors for pancreatic injury. Comorbidities included hypertension, diabetes mellitus, hyperlipidemia, congestive heart failure, and myocardial infarction. Baseline laboratory tests were reported for all patients before ICI therapy.

### Oncologic data

Oncologic details related to the type, site, and stage of cancer, as well as interruption of ICI therapy, were recorded. Malignancies were categorized as melanoma, solid tumors (including genitourinary, lung, gastrointestinal, head and neck, and other solid tumors), and hematologic malignancies. The stage of melanoma and solid tumors was assessed according to the American Joint Committee on Cancer Staging System 7th edition [29]. The stage of hematologic malignancies was not reported. ICI agents used were ipilimumab, nivolumab, pembrolizumab, atezolizumab, durvalumab, avelumab, and tremelimumab. ICI agents were categorized as (1) CTLA-4 monotherapy, (2) PD-1/L1 monotherapy, or (3) combination of both. The mean duration of ICI therapy and the median number of ICI doses were recorded. Reasons for ICI therapy discontinuation, permanent or temporary, were also documented.

### Pancreas-related data

Baseline lipase and amylase values were assessed for all patients to check for pancreatic injury prior to ICI therapy. We collected the peak value of serum lipase and amylase, as well as computed tomography (CT) findings of the pancreas (such as peripancreatic fat stranding, pancreatic enlargement with heterogeneous enhancement, and segmental hypoenhancement). We defined ICIPI as elevation of lipase with and without clinical presentation. Typical clinical presentation of acute pancreatitis was epigastric pain aggravated by leaning forward or radiating to the back, with or without associated nausea and vomiting. Other clinical signs and symptoms of ICIPI included fever, diarrhea, dyspnea, and hemodynamic instability (as a result of ICIPI or other reasons). Diarrhea was recorded as a part of ICIPI clinical presentation if it was not diagnosed previously as a symptom of immune-mediated colitis and was not of infectious cause. Thereafter, patients were divided into two groups on the basis of the presence of typical acute pancreatitis symptoms. Duration of clinical symptoms was measured from symptom onset to improvement.

Data relating to the treatment of pancreatic injury included use and amount of intravenous (IV) fluid, corticosteroids (either for pancreatitis only or concurrently for other reasons), and duration of treatment. Hospitalization to treat elevated serum lipase with IV fluids or/and steroids was also documented. Short-term adverse outcomes of ICIPI were reported as pancreatic pseudocyst and need for hospitalization (along with duration of hospitalization). Long-term adverse outcomes of pancreatitis included diabetes, chronic pancreatitis features on imaging (such as pancreatic atrophy), and recurrence of lipase elevation within a mean follow-up duration of 16 months.

### Statistical analysis

The distribution of continuous variables was summarized by means and standard deviations or medians and interquartile ranges. The distribution of categorical variables was summarized using frequencies and percentages. Continuous variables were compared between groups using the Wilcoxon rank-sum test. The Fisher exact test was conducted to evaluate associations between categorical variables. Univariate logistic regression analysis was conducted to assess for the risk of long-term adverse outcomes of ICIPI. Kaplan-Meier curves and the log-rank test were used to estimate and compare unadjusted overall survival time distributions. A multivariable Cox proportional hazards model was used to evaluate the ability of independent covariates to predict overall survival. All statistical evaluations were two-sided, and a *P* value of ≤0.05 was considered statistically significant. Statistical analysis was carried out using the SPSS Statistics software program (version 24.0; IBM Corporation, Armonk, NY).

## Results

### Patient characteristics

Among 5,762 patients who received ICI therapy during the period studied, 2,279 patients had lipase levels tested; 627 patients received anti-CTLA-4 monotherapy, 1434 received PD-1/L1 monotherapy, and 218 received combination therapy. In the CTLA-4 monotherapy group, 12 patients (2%) developed grade ≥ 3 serum lipase elevation that was deemed related to ICI therapy. Among patients who received PD-1/L1 monotherapy, 53 (4%) had ICIPI. In the combination therapy group, 17 (8%) developed ICIPI. Thus, our cohort included 82 patients. Baseline clinical characteristics of the patients are shown in Table 1. In our cohort, most patients (66%) were male with a mean age of 57 years. Melanoma was the most common malignancy in our cohort (37%). The median number of ICI doses was 4 (interquartile range 1–25). Other non-pancreatic irAEs reported at the time of ICIPI onset included entercolitis in 27 patients (33%), hepatic injury in 17 patients (21%), dermatologic events in 13 patients (16%), and endocrine events in 7 patients (9%).

**Table 1:** Clinical characteristics of patients in our cohort (*n* = 82)

Characteristic	No. of patients (%)
Mean age (standard deviation)	57 years (14)
Male sex	54 (66)
Race/ethnicity	
White	63 (77)
Black	7 (9)
Hispanic	9 (11)
Other	3 (4)
Baseline risk factors	
History of smoking	35 (43)
Alcohol consumption	35 (43)
Type II diabetes mellitus	21 (26)
Drug allergy	51 (62)
Prior history of pancreatitis	7 (9)
Pancreatic metastasis	11 (13)
Cancer type	
Melanoma	30 (37)
Genitourinary	24 (29)
Lung, head and neck	11 (13)
Gastrointestinal	5 (6)
Other solid tumors	5 (6)
Hematologic malignancies	7 (9)
Cancer stage^a^	
Stage III	3 (4)
Stage IV	72 (96)
Median number of immune checkpoint inhibitor doses (interquartile range)	4 (1-25)
Checkpoint inhibitor type	
CTLA-4 monotherapy	12 (15)
PD-1/L1 monotherapy	53 (65)
Combination therapy^b^	17 (21)
Other immune-related adverse events	
Gastrointestinal	27 (33)
Hepatic	17 (21)
Dermatologic	13 (16)
Endocrine	7 (9)
Pulmonary	5 (6)
Other	6 (7)

^a^Cancer stage was recorded for 75 patients. For solid tumors, the TNM system of the American Joint Committee on Cancer 7th edition was used for cancer staging. No cancer stage was record for hematologic cancer

^b^Combination of CTLA-4 and PD-1 or PD-L1 therapy

### Clinical characteristics of ICIPI

The median time from ICI initiation to peak lipase elevation was longer in patients who received PD-1/L1 monotherapy than in patients who received CTLA-4–based regimens (median 146 days for PD-1/L1 compared with 69 days for CTLA-4 monotherapy and 110 days for combination therapy, *P* = 0.033; Additional file 1: Table S1). Clinical symptoms of pancreatitis were epigastric pain in 32 patients (39%), nausea and vomiting in 23 (28%), fever in 7 (9%), and diarrhea in 16 (20%). Twenty-four patients had more than one of these symptoms. Forty five patients had completely asymptomatic presentation. Three of the patients who had completely asymptomatic presentation had CT findings suggestive of ICIPI with lipase elevation. No differences were observed in the clinical characteristics of patients according to ICI type. Typical pancreatitis presentation was observed in 32 patients (39%) and the other 50 (61%) did not have typical pancreatitis symptoms (Table 2). According to CTCAE grades for pancreatitis, 41 patients (50%) in our cohort had grade 2 pancreatitis (enzyme elevation or radiologic findings only) and the rest had grade 3 pancreatitis (pain, vomiting, medical intervention indicated). Fever occurred more frequently in patients with typical symptoms of pancreatitis than in those with asymptomatic lipase elevation (*P* = 0.013). Patients with a prior history of pancreatitis had an increased risk of ICIPI with clinical symptoms (*P* = 0.013). Although not statistically significant, CT findings suggestive of pancreatitis were more prevalent in patients who had symptoms of pancreatitis than in those who did not (*P* = 0.055). Patients with clinical symptoms of pancreatitis developed higher mean peak values of serum lipase than did patients without symptoms (3227 U/L for clinical symptoms compared with 1989 U/L for no symptoms, *P* = 0.032). There was no statistically significant difference in the peak values of serum amylase between the two groups (378 U/L for clinical symptoms compared with 270 U/L for no symptoms, *P* = 0.082).

**Table 2:** Clinical characteristics and outcomes stratified by clinical symptoms

Characteristic	Typical pancreatitis symptoms, *n* = 32	No typical pancreatitis symptoms, *n* = 50	*P*
Median time from immune checkpoint inhibitor initiation to peak lipase value (interquartile range)	107 days (3-511)	116 days (8-699)	0.520
Baseline risk factors			
History of smoking	14 (44)	21 (42)	1.000
Alcohol consumption	12 (38)	23 (46)	0.498
Type II diabetes mellitus	6 (19)	15 (30)	0.307
Drug allergy	22 (69)	29 (58)	0.360
Prior history of pancreatitis	6 (19)	1 (2)	0.013
Pancreatic metastasis	6 (19)	5 (10)	0.325
Mean biochemistry peak value (standard deviation)			
Lipase	3227 U/L (3563)	1989 U/L (1479)	0.032
Amylase	378 U/L (335)	270 U/L (217)	0.082
Computed tomography findings of pancreatitis	8 (25)	3 (6)	0.055
Hemodynamic instability			0.214
Pancreatitis only	5 (16)	2 (4)	
Other reasons	6 (19)	12 (24)	
Fever	6 (19)	1 (2)	0.013
Immune checkpoint inhibitor therapy discontinued	24 (75)	23 (46)	0.012
Treatment for pancreatitis			
Intravenous fluids	23 (72)	9 (18)	<0.001
Fluid amount^a^	3.9 L (1.4 L)	3.9 L (3.1 L)	0.995
Steroids			0.008
For pancreatitis only	9 (28)	2 (4)	
For other reasons also	5 (16)	15 (30)	
Mean time from peak lipase value to improvement to grade 1 (standard deviation)	55 days (54)	53 days (48)	0.877
Outcomes^b^	2 (6)	9 (18)	0.188
Pancreatic pseudocyst	2 (6)	1 (2)	0.557
Hospitalization	15 (47)	0 (0)	<0.001
Diabetes	1 (3)	1 (2)	1.000
Chronic pancreatitis features	1 (3)	2 (4)	1.000
Recurrence	1 (3)	3 (6)	1.000

^a^The amount of intravenous fluid administered within 48 hours after the onset of immune checkpoint inhibitor-induced pancreatic injury was recorded.

^b^Including short-term outcomes (pancreatic pseudocyst and hospitalization) and long-term outcomes (diabetes, chronic pancreatitis features, and recurrence)

ICI therapy was more often discontinued in patients who developed clinical symptoms than in patients without symptoms of pancreatitis (*P* = 0.012). Thirty two patients received IV fluids for ICIPI; most were normal saline (*n* = 28), followed by ½ normal saline (*n* = 3) and lactated ringer (*n* = 1). IV fluids were given more frequently in patients who developed clinical symptoms of pancreatitis than in patients who did not (*P* < 0.001). Patients who had clinical symptoms of pancreatitis received steroids and required hospitalization more frequently than patients who had no symptoms (*P* = 0.008 for steroid; *P* < 0.001 for hospitalization).

Eleven patients (13%) had pancreatic metastasis at the time of ICI initiation. Mean baseline lipase values were similar between patients who had pancreatic metastasis and those who did not (146 for those with metastasis and 162 for those with no metastasis, *P* = 0.769). We did not find any statistically significant differences in regards to epigastric pain (*P* = 0.325) and peak lipase value (*P* = 0.827) between patients who had pancreatic metastasis and those who did not. Also, there was no statistically significant difference in the prevalence of CT findings of pancreatitis (*P* = 0.238) and the treatment administered for pancreatitis (IV fluid, *P* = 0.325; steroids, *P* = 0.347) between patients who had pancreatic metastasis and those who did not. No significant associations were found between pancreatic metastasis and short-term or long-term adverse outcomes of ICIPI.

### Radiologic characteristics of pancreatic injury

Among the 64 patients who had CT scan evaluation, findings of pancreatitis were found in 11 patients (17%). The most common features suggestive of pancreatitis were segmental hypoenhancement, stranding in peripancreatic fat, and pancreatic enlargement with heterogeneous enhancement. Three of the patients who had CT findings of pancreatitis had also pancreatic metastasis, and the other eight did not. Additional file 2: Figure S1 shows features of pancreatitis with and without pancreatic metastasis.

### Short-term outcomes of ICIPI

In regards to short-term clinical outcomes of ICIPI, three patients (4%) developed a pancreatic pseudocyst and 18 (22%) required hospitalization with a mean duration of 5 days. The associations of steroids and IV fluids with short-term outcomes of ICIPI are summarized in Table 3. The use of steroids and IV fluids did not shorten the time from peak lipase value to improvement to grade 1 or below (*P* = 0.711 for steroids and *P* = 0.850 for IV fluids), the duration of symptoms (*P* = 0.824 for steroids and *P* = 0.650 for IV fluids), or the duration of hospitalization (*P* = 0.762 for steroids and *P* = 0.166 for IV fluids). The administration of steroids or IV fluids was not associated with ICI therapy discontinuation (*P* = 1.000 for steroids and *P* = 0.113 for IV fluids). Upon stratifying patients by the grade of lipase elevation, we found that steroid therapy did not affect the short-term outcomes in patients with grade 3 compared with grade 4 lipase elevation (Additional file 1: Table S2). Similarly, IV fluid administration was not associated with different short-term outcomes in patients with grade 3 or grade 4 lipase elevation (Additional file 1: Table S3).

**Table 3:** Short-term clinical outcomes of pancreatitis by treatment for immune checkpoint inhibitor-induced pancreatic injury

Outcome	Steroids, no. (%)			Intravenous fluids, no. (%)		
	Steroids, *n* = 31	No steroids, *n* = 51	*P*	Intravenous fluids, *n* = 32	No intravenous fluids, *n* = 50	*P*
Mean time from peak lipase value to improvement to grade 1^a^ (standard deviation)	55 days (11)	48 days (7)	0.711	55 days (51)	52 days (49)	0.850
Mean duration of symptoms^b^ (standard deviation)	5 days (3)	4 days (2)	0.824	4 days (3)	5 days (3)	0.650
Pseudocyst	1 (3)	2 (4)	1.000	3 (9)	0 (0)	0.056
Hospitalization	6 (19)	9 (18)	1.000	14 (44)	1 (2)	<0.001
Mean duration of hospitalization (standard deviation)	4 days (3)	5 days (2)	0.762	5 days (3)	1 day (-)	0.166
Intravenous fluids	14 (45)	18 (35)	0.484	-	-	-
Steroids	-	-	-	14 (44)	17 (34)	0.484
Immune checkpoint inhibitor therapy interrupted	18 (58)	29 (57)	1.000	22 (69)	25 (50)	0.113

^a^Improvement was defined as return of lipase value to grade 1.

^b^Duration of symptoms was measured for 35 patients with symptoms

### Long-term adverse outcomes of ICIPI

Long-term adverse outcomes of ICIPI were chronic pancreatitis in three patients (4%) and diabetes in six (7%); five of them required insulin and the sixth required metformin. Four of the patients who developed consequent diabetes did not receive steroids for ICIPI. Additionally, 4 of the 35 patients (11%) who resumed ICI therapy after ICIPI had recurrence of lipase elevation, one symptomatic and three asymptomatic. Patients who developed adverse outcomes of ICIPI received longer-duration ICI therapy than patients who did not (mean 412 days for adverse outcomes and 200 days for no adverse outcomes, *P* = 0.006; Table 4). IV fluids were more frequently given to patients without subsequent long-term adverse outcomes of ICIPI than patients with adverse outcomes (*P* = 0.044).

**Table 4:** Clinical characteristics of patients who had adverse outcomes of immune checkpoint inhibitor-induced pancreatic injury (chronic pancreatitis, diabetes, and recurrence)

Characteristic	No. (%)		*P*
	Adverse outcomes, *n* = 11	No adverse outcomes, *n* = 71	
Mean duration of ICI therapy (standard deviation)	412 days (361)	200 days (197)	0.006
Checkpoint inhibitor type			0.739
CTLA-4–based therapy^a^	3 (27)	26 (37)	
PD-1/L1 monotherapy	8 (73)	45 (63)	
Clinical presentation			
Epigastric pain	2 (18)	30 (42)	0.188
Nausea and vomiting	2 (18)	21 (30)	0.720
Fever	1 (9)	6 (8)	1.000
Dyspnea	0 (0)	17 (24)	0.109
Hemodynamic instability	1 (9)	24 (34)	0.159
Mean peak lipase value (standard deviation)	1700 U/L (636)	2592 U/L (2723)	0.285
Computed tomography findings of pancreatitis	1 (9)	10 (14)	1.000
Mean duration from peak lipase value to improvement to grade 1^b^ (standard deviation)	59 days (33)	53 days (53)	0.693
Immune checkpoint inhibitor therapy resumption	7 (64)	28 (39)	0.191
Treatment for pancreatitis treatment			
Hospitalization	1 (9)	14 (20)	0.679
Intravenous fluids	1 (9)	31 (44)	0.044
Fluid amount^c^	2.1 L (-)^d^	3.9 L (2.0)	0.382
Steroids	5 (45)	26 (37)	0.740

^a^CTLA-4–based therapy included CTLA-4 monotherapy and combination of CTLA-4 and PD-1/L1 agents.

^b^Improvement was defined as return of lipase value to grade 1.

^c^The amount of intravenous fluid administered within 48 hours after the onset of immune checkpoint inhibitor-induced pancreatic injury.

^d^Only one patient had data available

Results of logistic regression analysis of long-term adverse outcomes are shown in Table 5. Smoking history (odds ratio 4.35; 95% confidence interval 1.06–17.81, *P* = 0.041) and hyperlipidemia (odd ratio 6.15; 95% confidence interval 1.24–30.55, *P* = 0.026) were associated with an increased risk of long-term adverse outcomes of ICIPI. Patients who received IV fluids had a favorable ICIPI long-term outcomes profile (odds ratio 0.21; 95% confidence interval 0.06–0.79, *P* = 0.022). No significant associations were reported between long-term adverse outcomes of ICIPI and ICI type, symptoms of pancreatitis, abnormal CT findings, peak lipase and amylase values, and use of steroids.

**Table 5:** Univariate logistic regression analysis for long-term pancreatic adverse outcomes

Characteristic	Odds ratio (95% confidence interval)	*P*
Age	0.96 (0.92-1.02)	0.234
Male	2.60 (0.52-12.96)	0.244
Smoking	4.35 (1.06-17.81)	0.041
Alcohol	1.74 (0.48-6.24)	0.397
Hyperlipidemia	6.15 (1.24-30.55)	0.026
Prior history of pancreatitis	0.91 (0.10-8.24)	0.936
Pancreatic metastasis	0.61 (0.07-5.29)	0.654
CTLA-4–based regimen^a^	1.54 (0.38-6.32)	0.549
Continued immune checkpoint inhibitor therapy	2.69 (0.72-10.04)	0.141
Time from initiation of immune checkpoint inhibitors to onset	0.99 (0.99-0.99)	0.016
Pancreatic symptoms	2.02 (0.40-10.13)	0.393
Peak lipase value	1.00 (0.99-1.01)	0.274
Peak amylase value	1.00 (0.99-1.01)	0.404
Abnormal computed tomography findings	0.49 (0.06-4.31)	0.519
Intravenous fluids	0.21 (0.06-0.79)	0.022
Fluid resuscitation volume	0.57 (0.16-1.99)	0.380
Steroids	1.44 (0.40-5.19)	0.572
Hospitalization	0.32 (0.04-2.66)	0.291

^a^CTLA-4–based therapy included CTLA-4 monotherapy and combination of CTLA-4 and PD-1/L1 agents

Among the 82 patients, 18 received IV fluids without steroids, and none of these patients developed long-term adverse outcomes from ICIPI. Fourteen patients received both IV fluids and steroids, and among these patients, only one (7%) developed long-term adverse outcomes of ICIPI. On the other hand, four of the 17 patients (24%) who received steroids without IV fluids developed long-term adverse outcomes from ICIPI. Similarly, six of the 33 patients (18%) who received neither IV fluids nor steroids developed long-term adverse outcomes (Additional file 3: Figure S2A).

**Fig. 1:**
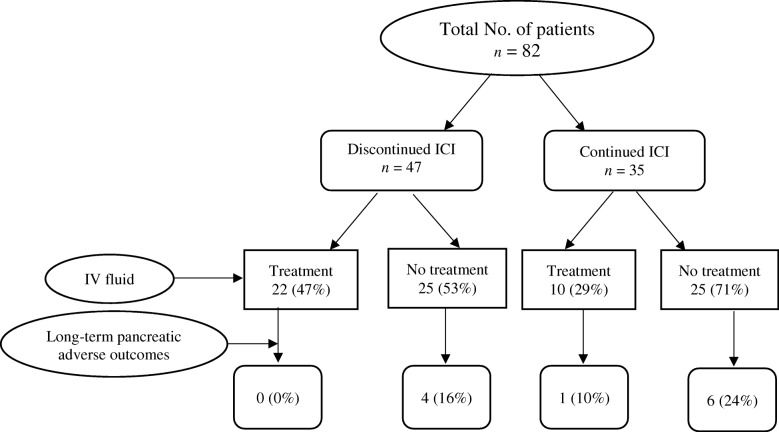
Long-term adverse outcomes of immune checkpoint inhibitor-induced pancreatic injury by treatment for pancreatitis

**Fig. 2:**
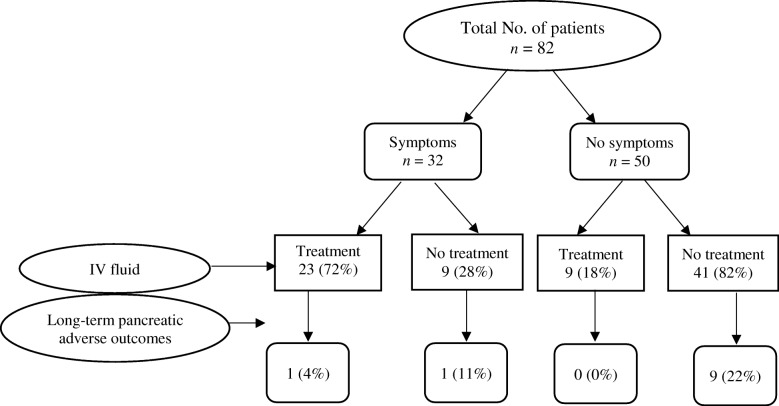
Long-term adverse outcomes of immune checkpoint inhibitor (ICI)-initiated pancreatic injury by discontinuation of ICI therapy and use of intravenous fluids

Only one (3%) of the 32 patients who received IV fluids developed long-term adverse outcomes of ICIPI, whereas 10 (20%) of the 50 patients who did not receive IV fluids developed long-term adverse outcomes. None of the patients who discontinued ICI therapy after ICIPI and received IV fluids had long-term adverse outcomes (Fig. 1). By contrast, 24% of the patients who continued ICI therapy and did not receive IV fluids had long-term adverse outcomes. Overall, patients who had no symptoms of pancreatitis and received no IV fluids had the highest rate of long-term adverse outcomes of ICIPI (22%) among all patients (Fig. 2). A total of 21 patients had no typical symptoms of pancreatitis, continued ICI therapy after ICIPI, and did not receive IV fluids. Of these, 6 (29%) had long-term adverse outcomes. Long-term outcomes of ICIPI stratified by the duration of follow-up is shown in Additional file 4: Figure S2B.

### ICIPI and overall survival

Kaplan-Meier curves showed that patients who resumed ICI therapy after ICIPI onset had slightly longer overall survival than did patients who did not, although this was not statistically significant (*P* = 0.0559; Additional file 5: Figure S3). Patients with long-term adverse outcomes of ICIPI had significantly longer overall survival duration than did patients without long-term adverse outcomes (*P* = 0.0295; Additional file 6: Figure S4). Overall survival rate did not differ between patients who received steroids and those who did not (*P* = 0.4088; Additional file 7: Figure S5) or between those who had symptoms of pancreatitis and those who did not (*P* = 0.1436; Additional file 8: Figure S6). The multivariable Cox model showed no significant associations between any of the clinical features and overall survival duration (Additional file 1: Table S4).

## Discussion

The current study represents by far the largest case series of patients with cancer who experienced ICIPI related to anti-CTLA-4 or anti-PD-1/L1 therapy. We found that this infrequent event can present as typical clinical symptoms of acute pancreatitis or it can be asymptomatic and present as an incidental finding. Correspondingly, ICIPI can present on CT imaging with features similar to those of traditional acute pancreatitis. In addition, ICIPI can lead to long-term adverse outcomes similar to those of acute pancreatitis. We found that the administration of IV fluids within 48 h after serum lipase elevation was associated with a reduced risk of long-term adverse outcomes. In contrast, corticosteroids had no significant benefit in shortening the acute phase of ICIPI, preventing long-term adverse outcomes from ICIPI, or improving overall survival rates. This observation remained the same when we stratified patients by the grade of lipase elevation. Furthermore, we found that ICI resumption after ICIPI led to statistically insignifciant longer overall survival durations.

Although ICIPI is uncommon and management procedures are generally not well defined, it is likely that the incidence of ICIPI will increase with the increasing use of ICIs, and thus awareness of this potential complication should be increased. Although any organ or tissue can be affected by irAEs, the dermatologic, gastrointestinal, hepatic, pulmonary, and endocrine systems are the most commonly affected [17]. We found that ICIPI was more common in patients with other adverse events. We therefore recommend that lipase values be obtained in patients who are diagnosed with non-pancreatic irAEs.

The incidence of ICIPI in our cohort was 2% for anti-CTLA-4 monotherapy, 4% for anti-PD-1/L1 monotherapy, and 8% for combination therapy. Our findings corroborate those of a meta-analysis showing that the incidence of ICIPI was 0.9–3% in patients receiving anti-CTLA-4 monotherapy, 1.2–2.1% in patients receiving combination therapy of anti-CTLA-4 and anti-PD-1, and 0.5–1.6% in patients receiving anti-PD-1 monotherapy [22]. Freeman-Keller et al. found that 1.35% of patients with resected and unresectable metastatic melanoma treated with nivolumab had asymptomatic grade 3 amylase/lipase elevation [30].

Clinical symptoms of pancreatitis were evident in 39% of patients with grade 3 or higher lipase elevation. The clinical presentation of ICIPI was similar to the clinical picture of traditional acute pancreatitis, including epigastric pain, nausea and vomiting, fever, and diarrhea. Our findings are consistent with those of a case series of 21 patients with ICI-induced serum lipase elevation, in which 14% of patients developed symptoms of pancreatitis and 86% of patients were asymptomatic [21]. No statistically significant differences were found in that study regarding the presence of clinical symptoms in patients who received anti-CTLA-4–based therapy compared with those who received anti-PD-1/L1 therapy. However, we found that patients who received anti-PD-1/L1 therapy developed elevated serum lipase later than did patients who received anti-CTLA-4–based therapy. Our findings are consistent a previous study showing that irAEs tended to present slightly later after anti-PD-1 therapy than after anti-CTLA-4 therapy [31].

Patients with clinical symptoms of pancreatitis had higher mean peak serum lipase values than did patients without clinical symptoms. This is not the case in patients with acute pancreatitis from other causes, where the level of serum lipase doesn’t correlate with the severity of pancreatic injury. Although elevated lipase values cannot determine the severity of pancreatitis, they do indicate an increased risk of pancreatitis [32]. According to our results, abnormal CT findings were more frequently found in patients who developed ICIPI with clinical symptoms than in patients without clinical symptoms. However, only 13% of our cohort had abnormal CT findings. This finding suggests that CT is not useful in the evaluation and management of ICIPI.

Pancreatic tumors are rarely the cause of acute pancreatitis [32]. However, whether pancreatic metastasis is associated with ICIPI and leads to acute pancreatitis is not clear. There are no data in the medical literature regarding the relationship between pancreatic metastasis and ICIPI. In our cohort, we assessed the effect of pancreatic metastasis on the clinical characteristics of ICIPI. Between those who had pancreatic metastasis and those who did not, we observed similar clinical presentation, mean peak value of lipase, CT findings of pancreatitis, treatment for ICIPI, and adverse outcomes of ICIPI.

Because of the lack of evidence and rare occurrence of ICIPI, the appropriate management of such events, which might lead to long-term adverse outcomes with an associated decrease in quality of life, has not been studied. Current guidelines on the evaluation and treatment of irAEs are very limited for ICIPI [27, 33]. Immunosuppressive therapy, such as steroids, is usually considered when managing irAEs [14, 17]. However, we did not find that steroids had any value in the treatment of ICIPI, in terms of preventing short-term and long-term adverse outcomes or improving overall survival. In addition, steroids might counteract the antitumor effects of ICIs, which are T-lymphocyte– mediated. Although a previous study suggested that steroids may improve clinical symptoms [16], this was not observed in our study.

The most prominent finding of our case series is the usefulness of aggressive IV fluid replacement in the treatment of ICIPI, which decreased the risk of long-term adverse outcomes of ICIPI, especially when ICI therapy was discontinued. This is part of the standard management of traditional acute pancreatitis, along with vital signs being closely monitored for any complications caused by pancreatitis [34]. Our study showed that patients with ICIPI who had clinical symptoms received IV fluids more frequently than those without clinical symptoms. This finding suggests that patients with asymptomatic ICIPI could have been undertreated owing to underestimation of the degree of ICIPI. This contradicts a previous recommendation to treat asymptomatic lipase elevation as an insignificant finding [21]. Our findings indicate that IV fluid administration should be considered, even in asymptomatic patients, for lipase elevation of grade 3 or higher, especially within 48 h of onset, to minimize long-term adverse outcomes. The presence of clinical symptoms may not correlate with the severity of ICIPI, but they may trigger earlier attention and evaluation. Nonetheless, according to CTCAE 4.03, pancreatic clinical symptoms are factors of the grade of pancreatitis; grade 3 pancreatitis presents with severe pain and vomiting and medical intervention is indicated, whereas grade 4 pancreatitis presents with life-threatening consequences and urgent intervention is indicated. Further prospective large-scale studies are needed to assess the significance of clinical symptoms in the management of ICIPI.

According to our findings, the resumption of ICI therapy was not associated with increased risk of long-term adverse outcomes of ICIPI. Additionally, overall survival was slightly longer in patients who resumed ICI therapy than in those who discontinued ICI therapy permanently. This finding could be confounded by the fact that patients who did not resume ICI might have had cancer progression that precluded them from receiving ICI therapy, and therefore, they had shorter survival duration. Michot et al. suggested withholding ICIs until lipase elevation is resolved and then resuming them or changing to an alternative anticancer therapy [21]. The findings of the current study support their recommendation that temporary ICI interruption followed by resumption once lipase values improve could potentially maximize the anticancer effect of ICIs.

Although the current study is the largest case series of patients with ICIPI, the study has certain limitations, mainly owing to its retrospective nature. First, our sample size was not adequate to perform further analysis to assess associations between ICIPI clinical outcomes and clinical features. Second, ICIPI consisted of both acute pancreatitis cases as well as asymptomatic lipase elevation. The extent of pancreatic injury in the latter could not be certainly depicted, and it could lead to an overestimation of pancreatic injury. Third, because evidence on the management of ICIPI in the medical literature is sparse, and no high quality evidence-based treatment guidelines are available for ICIPI, the treatment of ICIPI in our study was mainly at the discretion of the primary oncologist and based on the general clinical condition of the patient. Last, the cause of pancreatic injury is usually complex and involves numerous factors. Therefore, although we were able to exclude most of the causes that are reported to be associated with pancreatitis, we could not confirm that every case of pancreatic injury was related to ICI therapy.

## Conclusion

ICIPI, as an uncommon irAE, can present as typical acute pancreatitis in a subset of patients and may lead to the development of a pseudocyst and subsequent endocrine and exocrine insufficiency. In our series, the presence of symptoms of ICIPI was not an indicator of its severity or long-term adverse outcomes and overall survival. Regarding the management of ICIPI, IV fluid administration may decrease the risk of long-term adverse outcomes. In contrast, the benefit of steroids was not observed in preventing long-term adverse outcomes of ICIPI or improving overall survival. The resumption of ICI therapy after the improvement of grade 3 or higher lipase elevation appeared to have a low risk for morbidity and could potentially lead to favorable survival outcomes.

## Additional files

Additional file 1: **Table S1.** Patient clinical characteristics by type of immune checkpoint inhibitor therapy. **Table S2.** Short-term outcomes by grade of lipase elevation for patients who received steroids. **Table S3.** Short-term outcomes by the grade of lipase elevation for patients who received intravenous fluids. **Table S4.** Multivariable Cox regression for overall survival. (DOCX 16 kb)

Additional file 2: **Figure S1.** Computed tomography findings of ICIPI. (A) Images from a patient with pancreatic metastasis, demonstrating peripancreatic fat stranding indicating acute pancreatitis (short block arrows) and pancreatic duct dilation indicating metastasis in the pancreatic head (long arrow). (B) Images from a patient without pancreatic metastasis, demonstrating segmental hypoenhancement of the pancreatic head and proximal pancreatic body suggestive of acute pancreatitis (short block arrow), compared with normal enhancement of the distal pancreatic body and pancreatic tail (long arrow). (TIF 64274 kb)

Additional file 3: **Figure S2A.** Long-term adverse outcomes of immune checkpoint inhibitor-induced pancreatic injury by clinical symptoms of pancreatitis and use of intravenous fluids. (TIF 2184 kb)

Additional file 4: **Figure S2B.** Long-term adverse outcomes of immune checkpoint inhibitor-induced pancreatic injury by the median duration of follow-up and use of intravenous fluids. (TIF 2750 kb)

Additional file 5: **Figure S3.** Kaplan-Meier overall survival curves in patients who resumed and discontinued immune checkpoint inhibitor (ICI) therapy. (TIF 50634 kb)

Additional file 6: **Figure S4.** Kaplan-Meier overall survival curves in patients who did and did not have long-term adverse outcomes of immune checkpoint inhibitor-induced pancreatic injury. (TIF 49723 kb)

Additional file 7: **Figure S5.** Kaplan-Meier overall survival curves in patients who did and did not receive steroids for immune checkpoint inhibitor-induced pancreatic injury. (TIF 48223 kb)

Additional file 8: **Figure S6.** Kaplan-Meier overall survival curves in patients who did and did not have symptoms of pancreatitis with immune checkpoint inhibitor-induced pancreatic injury. (TIF 50469 kb)

## Abbreviations

CT: Computed tomography
CTCAE: Common Terminology for Adverse Events
CTLA-4: Cytotoxic T-cell lymphocyte-4
ICI: Immune checkpoint inhibitor
ICIPI: ICI-induced pancreatic injury
irAE: Immune-related adverse event
IV: Intravenous
PD-1: Programmed death-1
PD-L1: Programmed death ligand-1
