# Supplementary material for: Clinical characteristics and outcomes of immune checkpoint inhibitor-induced pancreatic injury
Source: J Immunother Cancer. 2019 Feb 6;7:31. doi: 10.1186/s40425-019-0502-7 (PMC6364483; doi:10.1186/s40425-019-0502-7)
Supplement: Supplementary file 1 — Table S1. Patient clinical characteristics by type of immune checkpoint inhibitor therapy. Table S2. Short-term outcomes by grade of lipase elevation for patients who received steroids. Table S3. Short-term outcomes by the grade of lipase elevation for patients who received intravenous fluids. Table S4. Multivariable Cox regression for overall survival. (DOCX 16 kb) [file 40425_2019_502_MOESM1_ESM.docx]

**Supp. Table 1.** Patient clinical characteristics by type of immune checkpoint inhibitor therapy.

| Characteristic | No. (%) | | | *P* |
| --- | --- | --- | --- | --- |
|  | **CTLA-4, n = 12** | **PD-1/L1, n = 53** | **Combination^a^, n = 17** |  |
| Clinical presentation |  |  |  |  |
| Epigastric pain | 5 (42) | 19 (36) | 8 (47) | 0.697 |
| Nausea and vomiting | 4 (33) | 15 (28) | 4 (24) | 0.844 |
| Fever | 1 (8) | 6 (11) | 0 (0) | 0.348 |
| Diarrhea | 2 (17) | 8 (15) | 6 (35) | 0.181 |
| Median time from immune checkpoint inhibitor initiation to peak lipase value (interquartile range) | 69 days (3-293) | 146 days (8-699) | 110 days (20-285) | 0.033 |
| Mean lipase peak value (standard deviation) | 3363 U/L (2514) | 2464 U/L (2856) | 1871 U/L (1154) | 0.306 |
| Immune checkpoint inhibitor therapy interrupted | 9 (75) | 29 (55) | 9 (53) | 0.404 |

^a^Combination of CTLA-4 and PD-1 or PD-L1 therapy.

**Supp. Table 2.** Short-term outcomes by grade of lipase elevation for patients who received steroids.

| Outcome | No. (%) | | *P* |
| --- | --- | --- | --- |
|  | **Grade 3, n = 15** | **Grade 4, n = 18** |  |
| Mean time from peak lipase value to improvement to grade 1^a^ (standard deviation) | 45 days (40) | 70 days (68) | 0.285 |
| Mean duration of symptoms^b^ (standard deviation) | 5 days (4) | 4 days (3) | 0.712 |
| Pseudocyst | 1 (7) | 0 (0) | 0.484 |
| Hospitalization | 4 (27) | 2 (11) | 0.394 |
| Mean duration of hospitalization (standard deviation) | 5 days (4) | 3 days (1) | 0.368 |
| Intravenous fluids | 8 (53) | 6 (33) | 0.479 |
| Immune checkpoint inhibitor therapy interrupted | 8 (53) | 10 (56) | 0.722 |

^a^Improvement was defined as return of lipase value to grade 1.

^b^Duration of symptoms was measured for 35 patients with symptoms.

**Supp. Table 3.** Short-term outcomes by the grade of lipase elevation for patients who received intravenous fluids.

| Outcome | No. (%) | | *P* |
| --- | --- | --- | --- |
|  | **Grade 3, n = 19** | **Grade 4, n = 30** |  |
| Mean time from peak lipase value to improvement to grade 1^a^ (standard deviation) | 52 (61) | 59 (58) | 0.777 |
| Mean duration of symptoms^b^ (standard deviation) | 4 (3) | 4 (3) | 0.807 |
| Pseudocyst | 2 (11) | 1 (3) | 0.552 |
| Hospitalization | 6 (31) | 8 (27) | 1.000 |
| Mean duration of hospitalization (standard deviation) | 5 (3) | 5 (2) | 0.864 |
| Steroids | 8 (42) | 6 (20) | 0.149 |
| Immune checkpoint inhibitor therapy interrupted | 8 (42) | 14 (47) | 0.699 |

^a^Improvement was defined as return of lipase value to grade 1.

^b^Duration of symptoms was measured for 35 patients with symptoms.

**Supp. Table 4.** Multivariable Cox regression for overall survival.

| Characteristic | HR (95% CI) | *P* |
| --- | --- | --- |
| Symptoms of pancreatitis | 1.82 (0.89-3.71) | 0.101 |
| Peak lipase value | 1.00 (1.00-1.00) | 0.916 |
| Peak amylase value | 1.00 (0.99-1.00) | 0.319 |
| ICI therapy continued | 0.61 (0.29-1.28) | 0.192 |
| Steroids administered | 0.73 (0.34-1.55) | 0.416 |
| Long-term adverse outcomes of ICI-induced pancreatic injury | 0.17 (0.02-1.23) | 0.079 |

Abbreviations: HR, hazard ratio; CI, confidence interval; ICI, immune checkpoint inhibitor.
