# Supplementary figures and images for: Clinical characteristics and outcomes of immune checkpoint inhibitor-induced pancreatic injury
Source: J Immunother Cancer. 2019 Feb 6;7:31. doi: 10.1186/s40425-019-0502-7 (PMC6364483; doi:10.1186/s40425-019-0502-7)

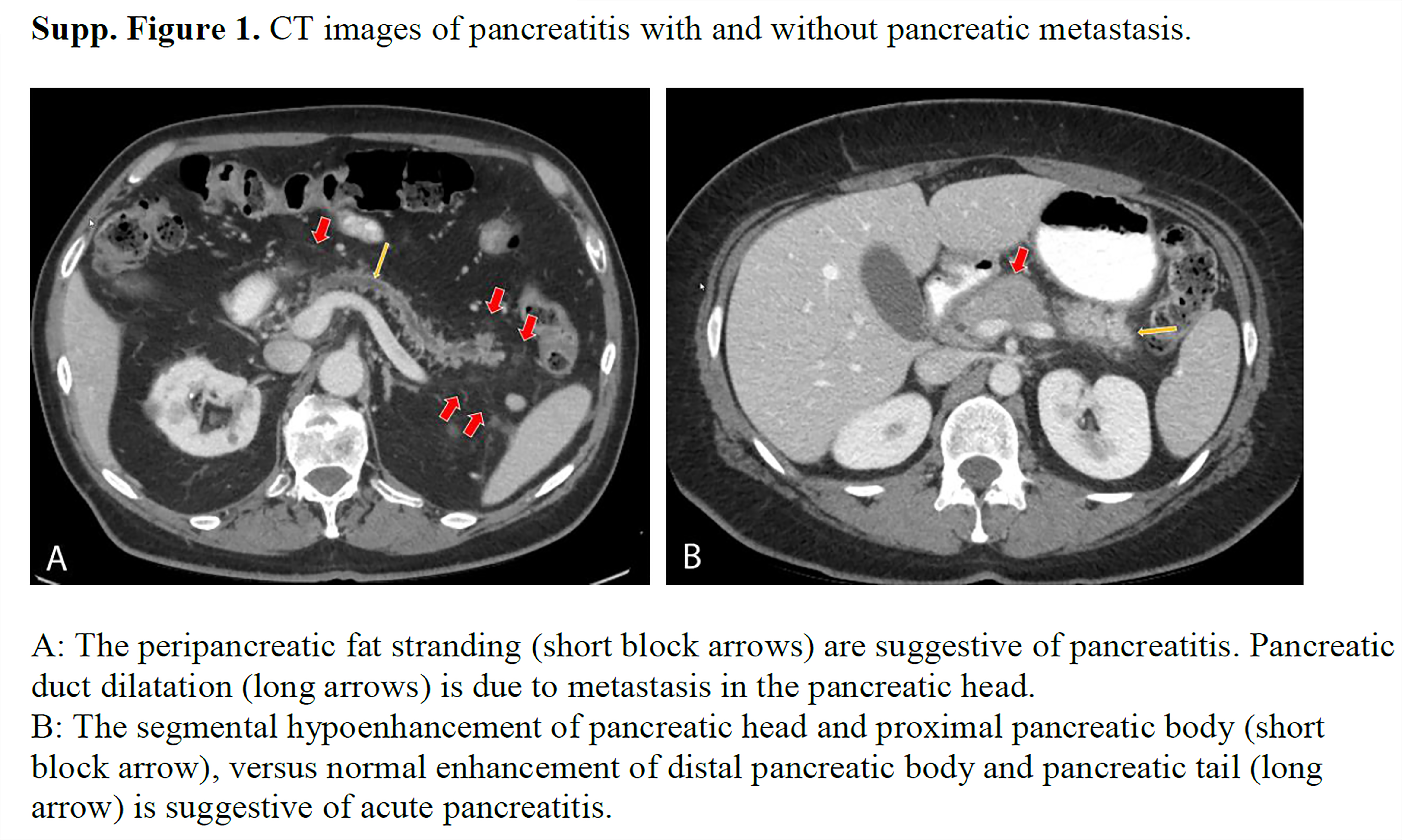

Supplement: Supplementary file 2 — Figure S1. Computed tomography findings of ICIPI. (A) Images from a patient with pancreatic metastasis, demonstrating peripancreatic fat stranding indicating acute pancreatitis (short block arrows) and pancreatic duct dilation indicating metastasis in the pancreatic head (long arrow). (B) Images from a patient without pancreatic metastasis, demonstrating segmental hypoenhancement of the pancreatic head and proximal pancreatic body suggestive of acute pancreatitis (short block arrow), compared with normal enhancement of the distal pancreatic body and pancreatic tail (long arrow). (TIF 64274 kb) [file 40425_2019_502_MOESM2_ESM.tif]

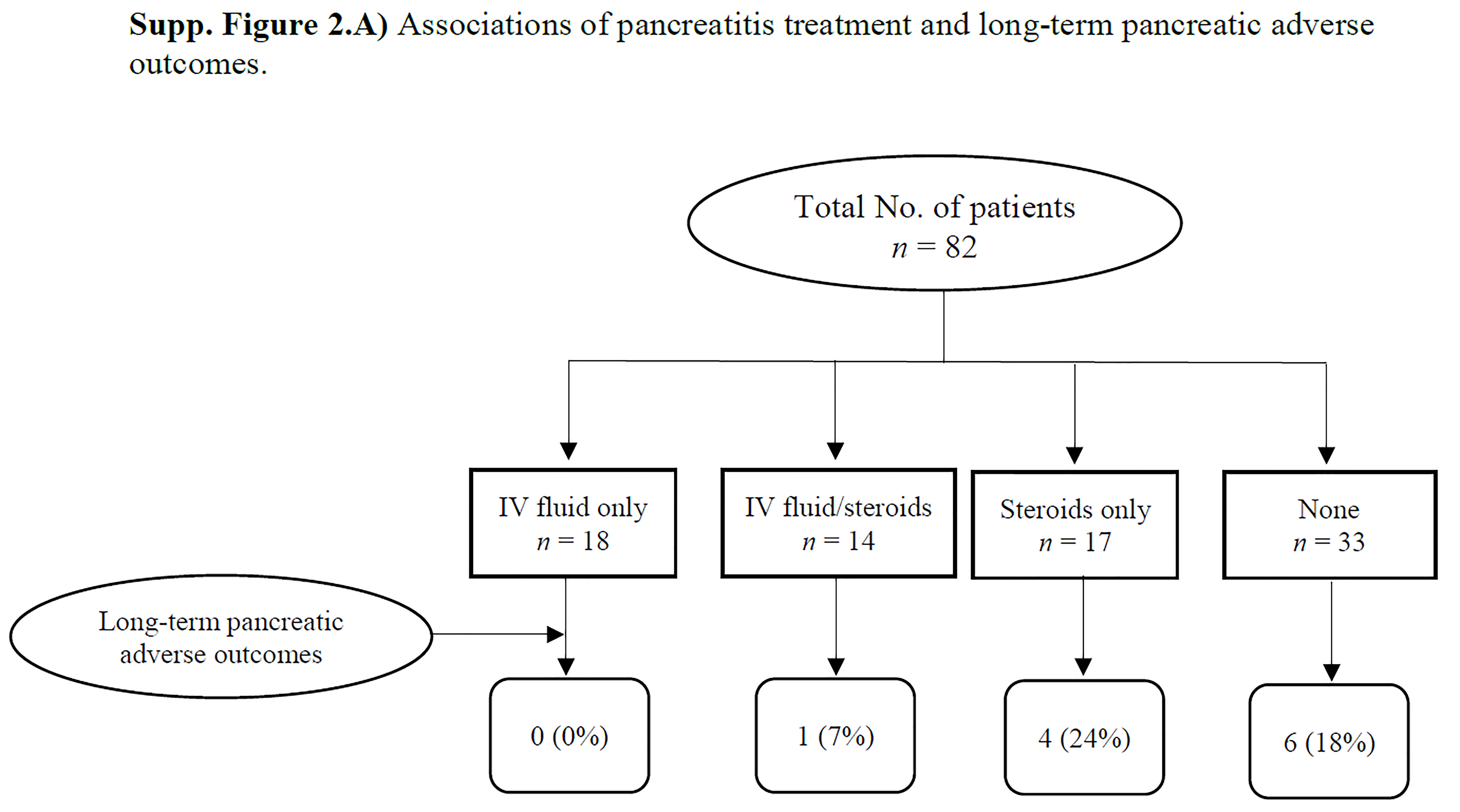

Supplement: Supplementary file 3 — Figure S2A. Long-term adverse outcomes of immune checkpoint inhibitor-induced pancreatic injury by clinical symptoms of pancreatitis and use of intravenous fluids. (TIF 2184 kb) [file 40425_2019_502_MOESM3_ESM.tif]

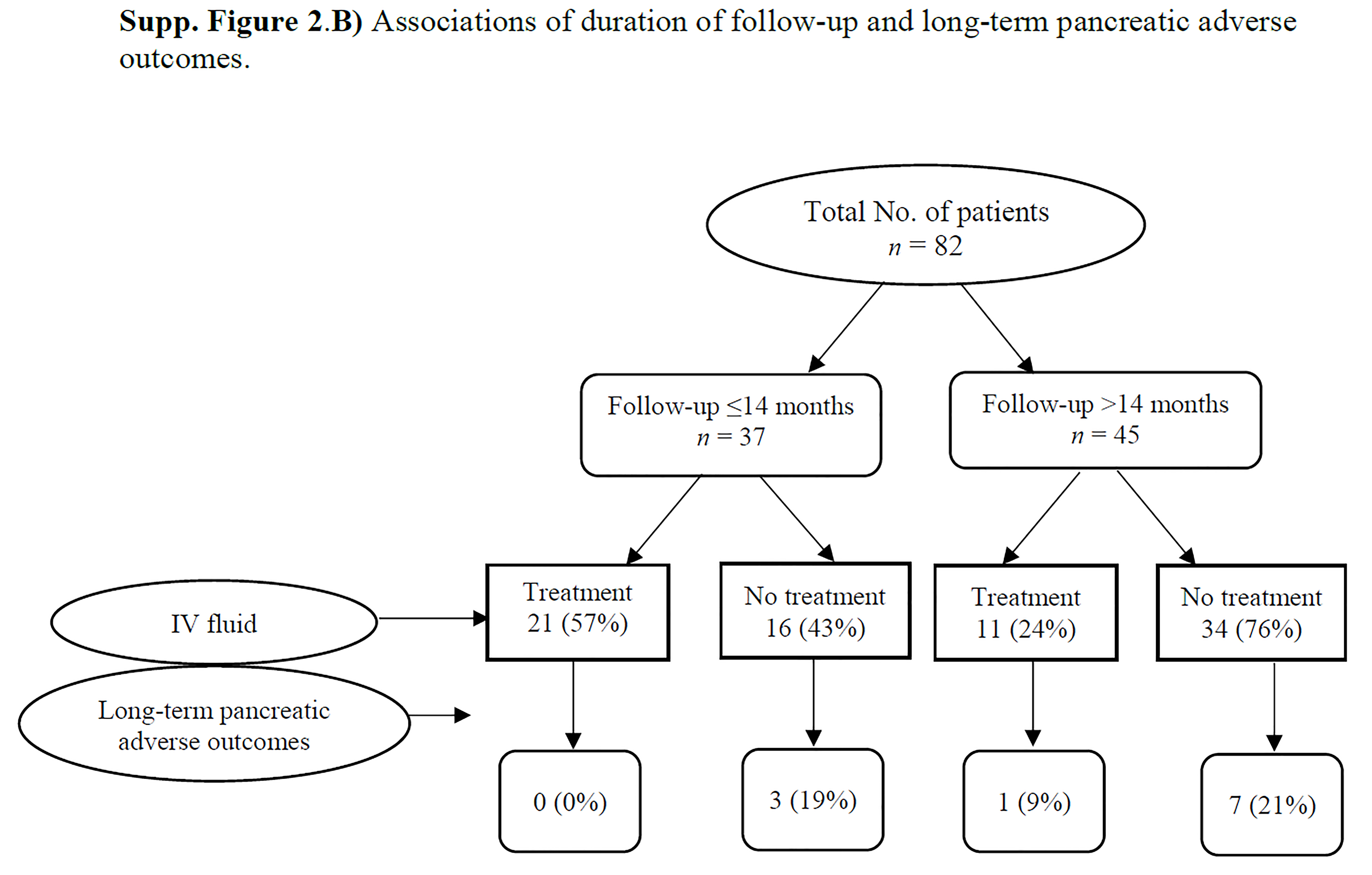

Supplement: Supplementary file 4 — Figure S2B. Long-term adverse outcomes of immune checkpoint inhibitor-induced pancreatic injury by the median duration of follow-up and use of intravenous fluids. (TIF 2750 kb) [file 40425_2019_502_MOESM4_ESM.tif]

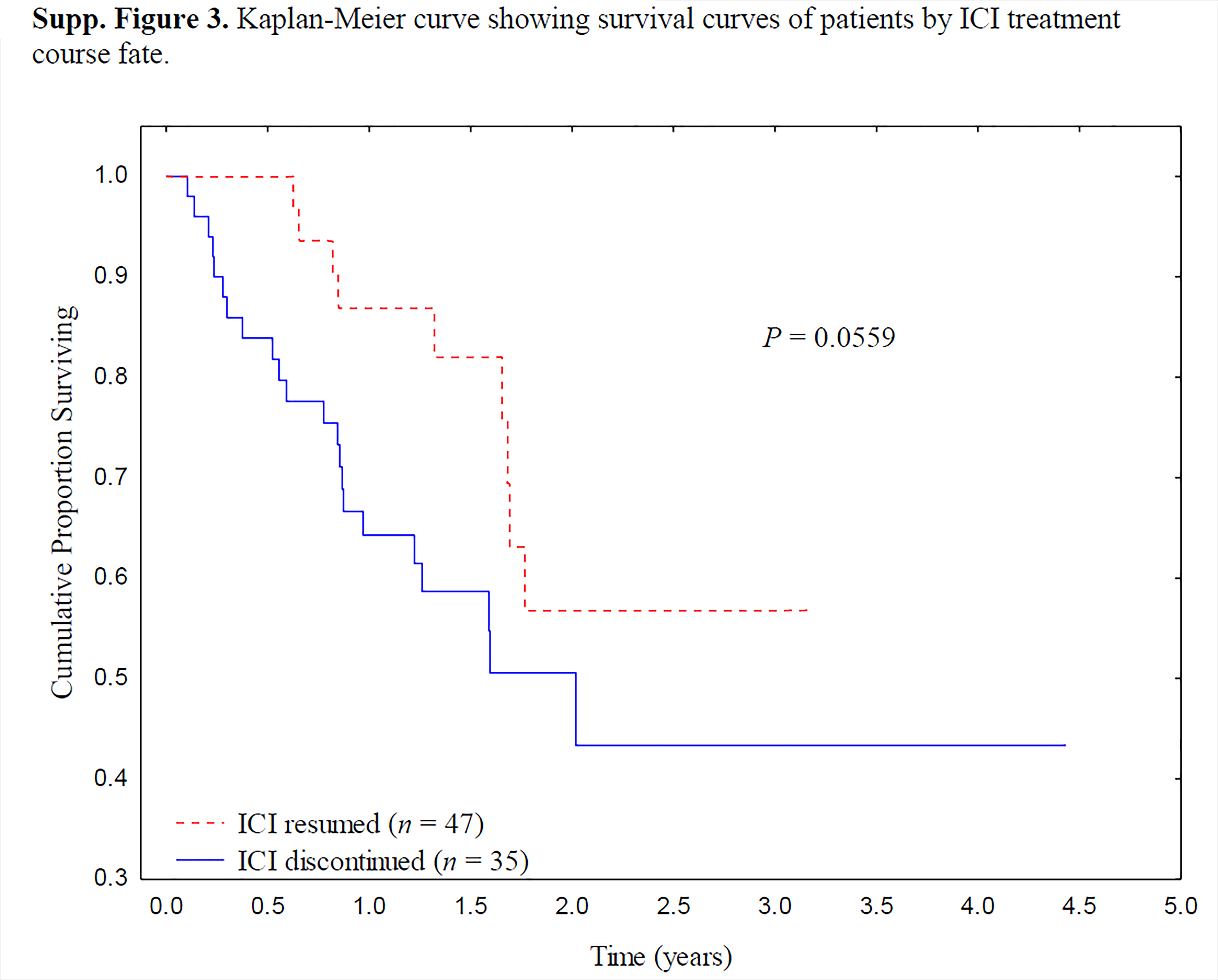

Supplement: Supplementary file 5 — Figure S3. Kaplan-Meier overall survival curves in patients who resumed and discontinued immune checkpoint inhibitor (ICI) therapy. (TIF 50634 kb) [file 40425_2019_502_MOESM5_ESM.tif]

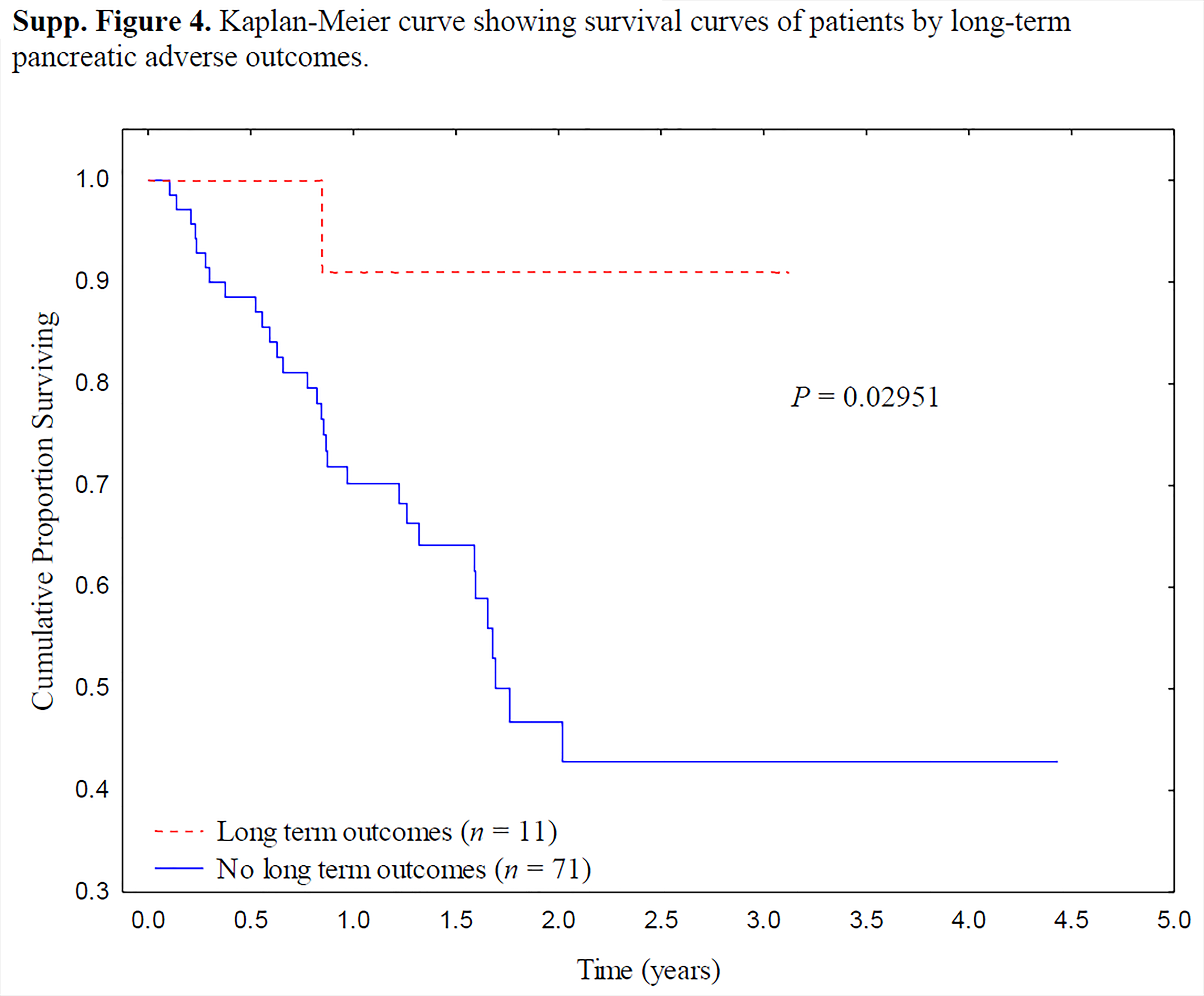

Supplement: Supplementary file 6 — Figure S4. Kaplan-Meier overall survival curves in patients who did and did not have long-term adverse outcomes of immune checkpoint inhibitor-induced pancreatic injury. (TIF 49723 kb) [file 40425_2019_502_MOESM6_ESM.tif]

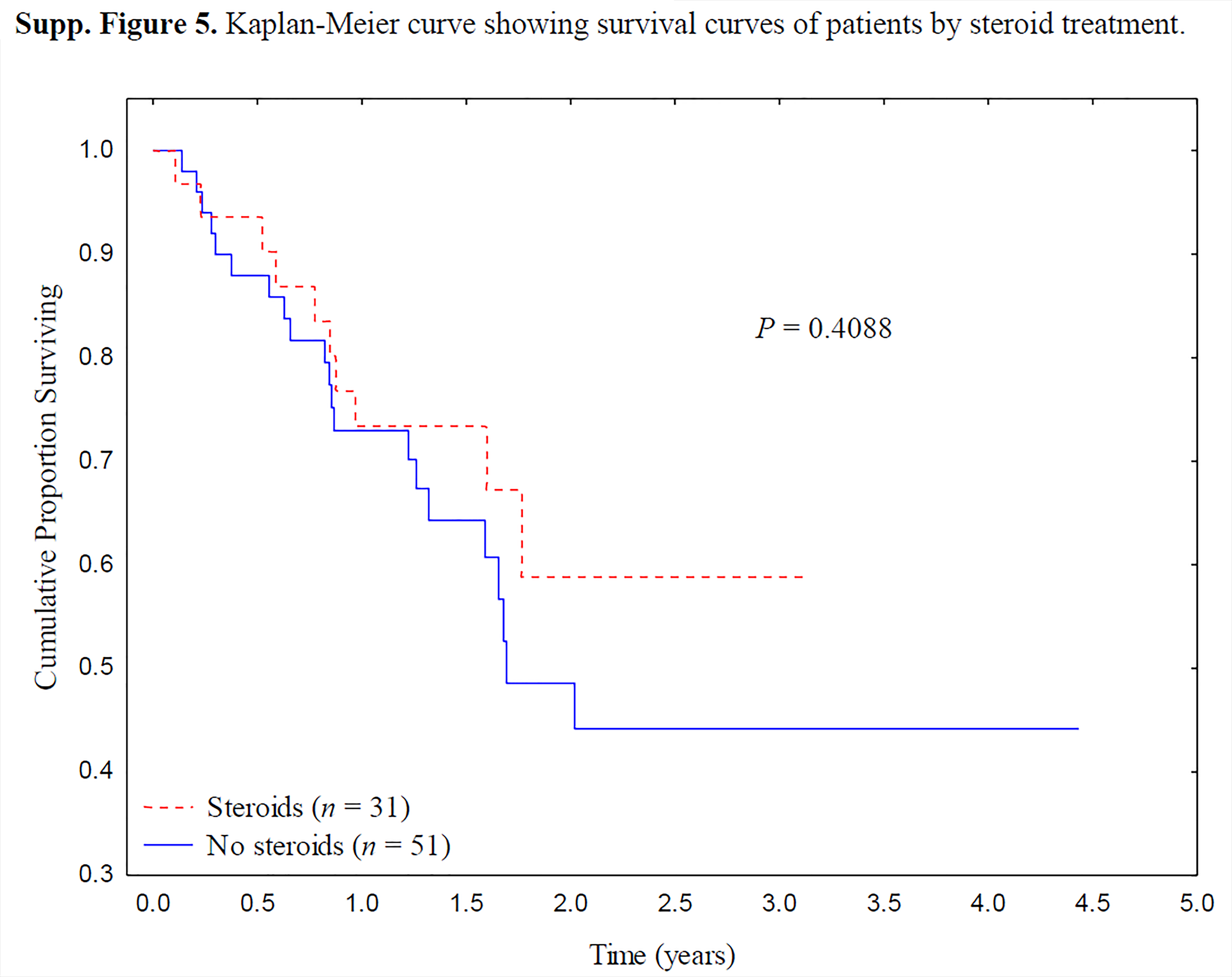

Supplement: Supplementary file 7 — Figure S5. Kaplan-Meier overall survival curves in patients who did and did not receive steroids for immune checkpoint inhibitor-induced pancreatic injury. (TIF 48223 kb) [file 40425_2019_502_MOESM7_ESM.tif]

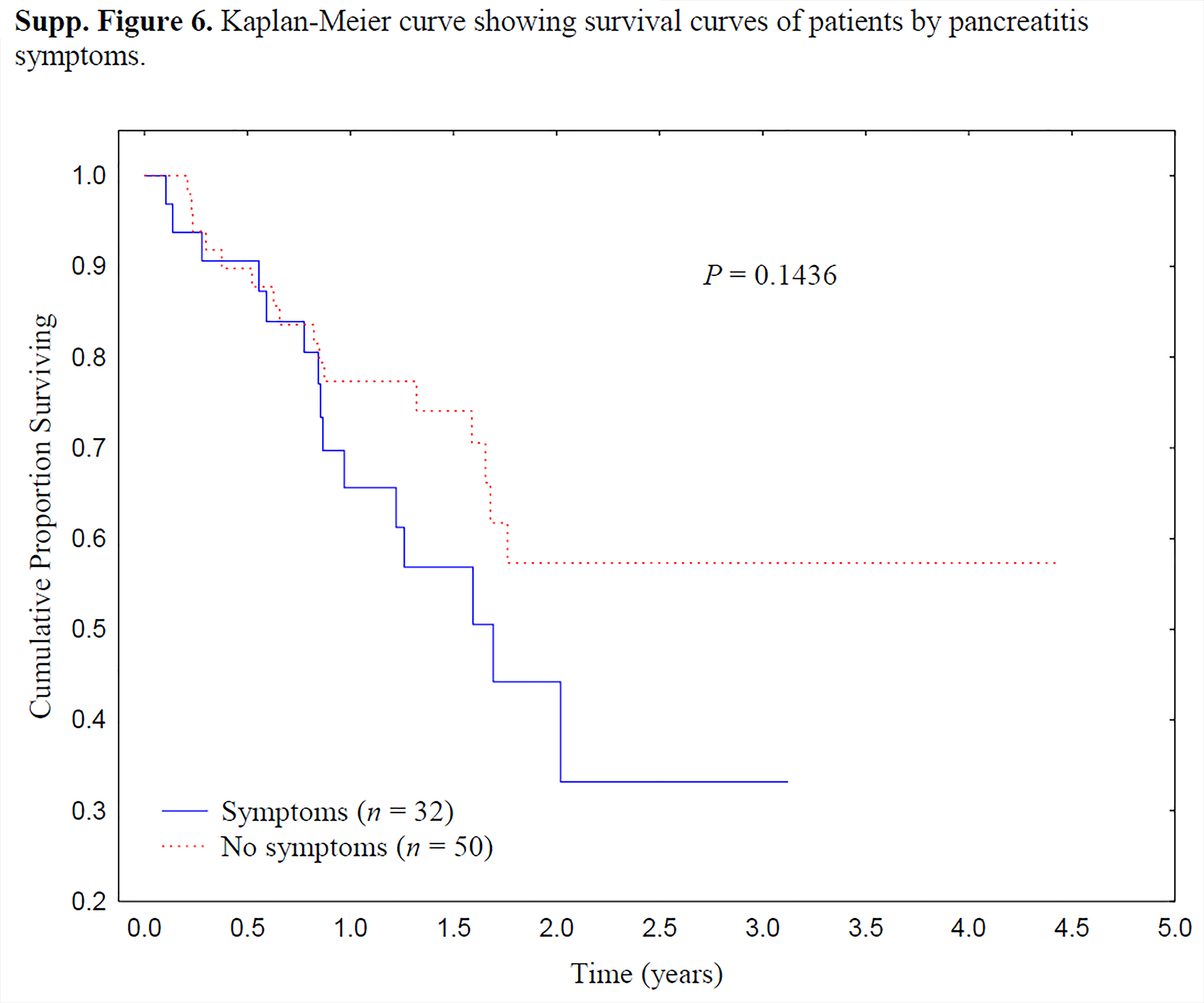

Supplement: Supplementary file 8 — Figure S6. Kaplan-Meier overall survival curves in patients who did and did not have symptoms of pancreatitis with immune checkpoint inhibitor-induced pancreatic injury. (TIF 50469 kb) [file 40425_2019_502_MOESM8_ESM.tif]
